# Chemical Composition, Antioxidant, and Anti-Inflammatory Activity of the Antarctic Lichen *Leptogium puberulum*: A Combination of Metabolomic, In Vitro, and In Silico Approaches

**DOI:** 10.3390/ijms27114822

**Published:** 2026-05-27

**Authors:** Alfredo Torres-Benítez, José Erick Ortega-Valencia, Juan Rodrigo Salazar, Katherine Monje, Jaqueline Ley-Martínez, Gabriel Vargas-Arana, Mario J. Simirgiotis

**Affiliations:** 1 Carrera de Química y Farmacia, Facultad de Ciencias, Universidad San Sebastián, General Lagos 1163, Valdivia 5090000, Chile; 2 Facultad de Ingeniería y Ciencias, Tecnológico Nacional de México/Instituto Tecnológico Superior de Álamo Temapache, Km. 6.5, Carr. Potrero del Llano-Tuxpan, Álamo Temapache C.P. 92730, Veracruz, Mexico; jortegavalencia244@gmail.com; 3 Chemistry Laboratory, Escuela Preparatoria Unidad Benjamín Franklin, Universidad La Salle, Av. Benjamín Franklin 45, Col. Condesa, Mexico City C.P. 06140, Mexico; juan.rodrigo.salazar@gmail.com; 4 Instituto de Farmacia, Facultad de Ciencias, Universidad Austral de Chile, Campus Isla Teja, Valdivia 5090000, Chile; katherine.monje@alumnos.uach.cl; 5 Instituto de Química Aplicada, Universidad Veracruzana, Luis Castelazo sin número, Colonia Industrial Animas, Xalapa C.P. 91190, Veracruz, Mexico; hackeol@hotmail.com; 6 Facultad de Industrias Alimentarias, Universidad Nacional de la Amazonía Peruana, Iquitos 16001, Peru; gabriel.vargas@unapiquitos.edu.pe

**Keywords:** Antarctica, extract, bioactive compounds, metabolomics, biological potential, pharmacokinetic properties, molecular docking, COX-2 inhibition

## Abstract

Lichenized fungi are a source of secondary metabolites with multiple biological potential. The objective of the study was to determine the chemical composition, antioxidant, and anti-inflammatory activity of the hydroalcoholic extract of the Antarctic lichen *Leptogium puberulum* through metabolomic, in vitro, and in silico analyses. Seventeen compounds were tentatively identified using UHPLC-ESI-QToF-MS. The phenolic composition yielded 6.356 mg GAE/g, and antioxidant activity assays showed IC_50_ values for DPPH• and ABTS•^+^ of 1187.149 and 207.00 µg/mL, respectively, along with 15.845 µmol Trolox/g for ORAC and 21.925 µmol Trolox/g for FRAP. The in silico evaluation was performed using OSIRIS Data Warrior, ProTox 3.0, and SwissTargetPrediction, identifying 9,10,12,13,14-pentahydroxytetracosanoic acid (PHTA), 9,10,12,13-tetrahydroxytricosanoic acid (THTA), 9,10,12,13-tetrahydroxyheneicosanoic acid (THHA), and 9,10,12,13-tetrahydroxydocosanoic acid (THDA) as the most promising compounds. These metabolites showed favorable pharmacokinetic properties, with no anticipated toxicological risks. Subsequently, their affinity for the cyclooxygenase-2 (COX-2) enzyme was evaluated by molecular docking with AutoDock Vina software version 1.2.3, and the most stable protein–ligand complexes were analyzed to characterize key interactions within the active site and subjected to molecular dynamics simulations with YASARA software version 19.1.27 for 100 ns. Overall, these results indicate that selected metabolites from *L. puberulum* may act as potential COX-2 inhibitors, supporting their relevance as lichen-derived anti-inflammatory agents and warranting further pharmacological investigation.

## 1. Introduction

In recent years, chronic noncommunicable diseases—including cardiovascular diseases, various types of cancer, metabolic disorders, and neurodegenerative diseases—have emerged as one of the leading causes of morbidity and mortality worldwide. Their development and progression are closely linked to oxidative stress and chronic inflammation, two interdependent biological processes that significantly contribute to cellular damage and tissue dysfunction [1].

Oxidative stress, resulting from an imbalance between the production of reactive oxygen species and the endogenous antioxidant capacity, triggers lipid peroxidation, DNA damage, and the activation of pro-inflammatory pathways. Added to this are enzymes such as NADPH oxidase and inducible nitric oxide synthase, which generate free radicals that amplify cellular damage, reinforcing a pathological cycle in which excess ROS stimulates the production of inflammatory mediators, and, in turn, inflammation increases ROS generation [1,2,3].

This sustained pro-oxidative and pro-inflammatory environment has been specifically associated with the progression of neurodegenerative diseases (e.g., Alzheimer’s disease and Parkinson’s disease), cardiovascular disorders, and type 2 diabetes [4]. In this context, the overexpression of cyclooxygenase-2 (COX-2) constitutes a critical relationship between oxidative stress and inflammation, since the COX-2-derived prostaglandin E2 (PGE2) activates prostaglandin receptors (EP1-EP4) that amplify both ROS production and inflammatory signaling [5].

Given the need to identify new therapeutic agents capable of modulating these inflammatory and oxidative pathways, bioactive compounds derived from the microorganisms, animal, plant, and fungal groups have gained significant importance and are a major focus of research [6]. One such group is lichens, which, due to their symbiotic nature and cosmopolitan distribution, are an important source of secondary metabolites with multiple biological activities [7,8].

Lichens are defined as organisms formed by a stable association between a fungus, or mycobiont—mostly belonging to the phylum Ascomycota and, to a lesser extent, to Basidiomycota—and a photosynthetic organism, or phycobiont, such as green algae or cyanobacteria. In this mutualistic relationship, the phycobiont provides carbohydrates derived from photosynthesis, while the mycobiont offers structural protection and facilitates the uptake of water and mineral nutrients. Due to their adaptability, they are widely distributed across all continents, with their presence determined by environmental factors such as climate and habitat characteristics. Currently, it is estimated that there are approximately 20,000 described species [9].

The genus *Leptogium* belongs to the family Collemataceae, comprises more than 100 species, and has a cosmopolitan distribution, particularly in tropical regions such as Africa, Asia, and South America [10,11,12]. Chemical studies of the genus are scarce and have focused primarily on secondary metabolites of endolichen fungi, such as *Epicoccum nigrum* in *L. masiaticum* [13], and on determining the lipid content in *L. phyllocarpum* and species of the genera *Cladonia*, *Cladia*, *Parmotrema*, *Ramalina*, *Cetraria*, and *Dictyonema glabratum*, currently known as *Cora glabrata* [14]. In Antarctica, phylogenetic analyses have identified endemic species that form unique lineages, such as *L. puberulum*, *Catillaria corymbosa*, *Himantormia lugubris*, *Pertusaria pertusa*, *Rhizoplaca aspidophora*, and *Umbilicaria antarctica*, along with others possessing more complex phenotypic and molecular characteristics, such as *Stereocaulon alpinum*, *Physcia caesia*, *Usnea aurantiacoatra*, and *Cladonia* spp. [15].

**Figure 1:**
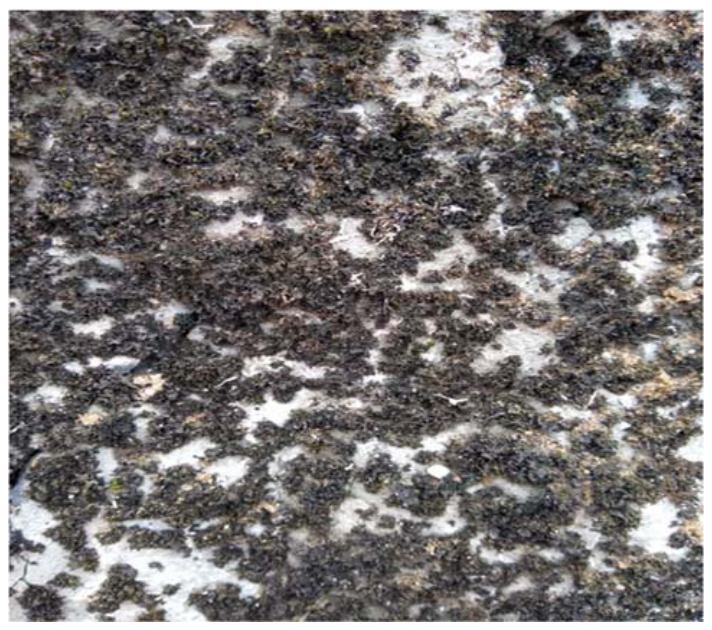
Lichen thallus of *L. puberulum*.

**Figure 2:**
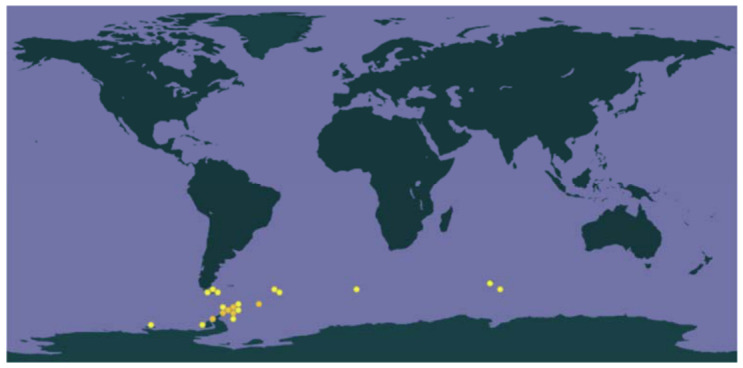
Distribution of *L. puberulum* in the Southern Hemisphere (GBIF).

In studies of lichens in the Antarctic region, one of the species of interest is *Leptogium puberulum* Hue, a lichen with a leafy thallus that is thin, up to 0.1 mm thick and 4 cm in diameter; it is brown when moist and dark olive green when dry; the lobes are irregular, densely curled, and the apices may be attached to the substrate or ascending; its underside may be smooth, bare, or covered with a fine tomentum, and apothecia are absent [16] (Figure 1). Ecologically, this species is strictly saxicolous and is associated with large and small rocks or gravel. Its distribution is limited to the Southern Hemisphere, specifically in areas of the Antarctic Peninsula, the South Shetland Islands in Maritime Antarctica, Magallanes in Chile, Tierra del Fuego in Chile and Argentina, the South Georgia Islands, the Falkland Islands, and other oceanic islands (Figure 2). About studies of biological processes, the only reported research involves an assessment of the functional diversity of the microbial community on the surface and inside the lichen thallus, which varies depending on the lichen’s growth site [17].

Based on the known effects and mechanisms of action of metabolites found in Antarctic lichens, it is hypothesized that the hydroalcoholic extract of *L. puberulum* exhibits antioxidant activity and that certain phenolic or lipid compounds demonstrate anti-inflammatory potential through the inhibition of the enzyme COX-2.

Accordingly, the objective of the study was to determine the chemical composition, as well as antioxidant and anti-inflammatory activity of the hydroalcoholic extract of the Antarctic lichen *L. puberulum*, through metabolomic, in vitro, and in silico analyses.

## 2. Results and Discussion

### 2.1. Chromatographic Analysis Through UHPLC-ESI-QToF-MS

**Figure 3:**
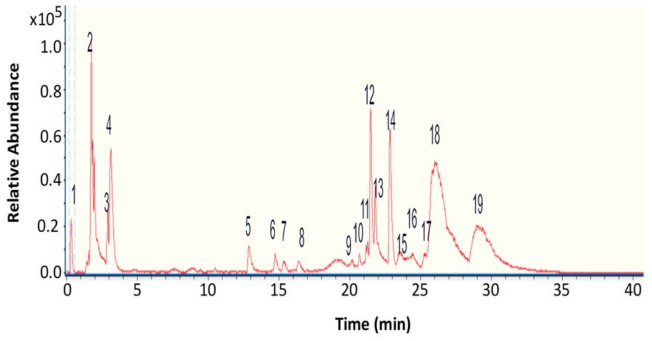
Chromatogram of the hydroalcoholic extract of *L. puberulum* obtained through UHPLC-ESI-QToF-MS.

The chemical profile of the hydroalcoholic extract of the Antarctic lichen *L. puberulum* was determined using state-of-the-art UHPLC-ESI-QToF-MS chromatography in negative ionization mode, as it provides greater sensitivity and clearer, more stable, and more abundant fragmentation patterns. The analysis tentatively identified the presence of 17 compounds classified as aromatic compounds, organic acids, and oxygenated lipids (Figure 3 and Table 1).

Carbohydrate: One carbohydrate was tentatively identified in the peak 2 as mannitol (C_6_H_13_O_6_).

Organic Acid: One organic acid was tentatively identified in the peak 3 as citric acid (C_6_H_7_O_7_).

Aromatics: Six aromatics were tentatively identified: the peak 4 as vaccihein A (C_18_H_17_O_9_) with a molecular anion at *m*/*z* 377.0814, peak 5 as benzoic acid (C_7_H_5_O_2_) with a molecular anion at *m*/*z* 121.0271, peak 7 as sinapoyltartronic acid (C_14_H_13_O_9_) with a molecular anion at *m*/*z* 323.04637 and diagnostic peaks at *m*/*z* 187.09352, 204.02216 and 160.03604, peak 8 as wedelolactone (C_16_H_9_O_7_) with a molecular anion at *m*/*z* 313.0308, peak 15 as phenylethyl primeveroside (C_19_H_27_O_10_) with a molecular anion at *m*/*z* 415.1654 and diagnostic peaks at *m*/*z* 297.2365 and 163.0348, and peak 18 as gleditschiaside A (C_21_H_27_O_11_) with a molecular anion at *m*/*z* 455.1558 and diagnostic peaks at *m*/*z* 409.0848 and 327.24549.

Fatty Acids: Nine fatty acids were tentatively identified as azelaic acid (C_9_H_15_O_4_, peak 6, molecular anion at *m*/*z* 187.0968 and diagnostic peaks at *m*/*z* 179.0330 and 125.0976), 1,3-diaceto-2-stearin (C_23_H_45_O_6_, peak 9 and a molecular anion at *m*/*z* 433.3144), 9,10,12,13-tetrahydroxyheneicosanoic acid (C_21_H_41_O_6_, peak 10 and a molecular anion at *m*/*z* 389.2892), 9,10,12,13,14-pentahydroxytetracosanoic acid (C_24_H_47_O_7_, peak 11, molecular anion at *m*/*z* 447.3306 and diagnostic peaks at *m*/*z* 389.2891, 429.3199 and 361.2581), 9,10,12,13-tetrahydroxydocosanoic acid (C_22_H_43_O_6_, peak 12, molecular anion at *m*/*z* 403.3047 and diagnostic peaks at *m*/*z* 385.2939 and 215.1273), pentahydroxyoxohexacosanoic acid (C_26_H_49_O_8_, peak 13, molecular anion at *m*/*z* 489.3403 and diagnostic peaks at *m*/*z* 403.3001 and 979.6848), 9,10,12,13-tetrahydroxytricosanoic acid (C_23_H_45_O_6_, peak 14 and a molecular anion at *m*/*z* 417.3204), methyl 9,10,11,12,13-pentahydroxy-14-oxoheptacosanoate (C_28_H_53_O_8_, peak 16, molecular anion at *m*/*z* 517.3719 and diagnostic peaks at *m*/*z* 457.3510 and 439.340), and pentahydroxyhexacosanoic acid (C_26_H_51_O_7_, peak 17 and a molecular anion at *m*/*z* 475.3618).

Additionally, one compound was identified as unknown.

The chemical composition detected in the hydroalcoholic extract of *L. puberulum* is consistent with previous studies on other Antarctic lichen species, such as *Lecania brialmontii*, *Pseudephebe pubescens*, *Sphaerophorus globosus* [18], *Cladonia gracilis*, and *Cladonia chlorophaea* [19], *Ochrolechia frigida*, *Placopsis contortplicata*, *Umbilicaria antarctica* [20], *Psoroma antarcticum*, *Psoroma hynorum* [21], *Gondwania regalis* [22], *Himantormia lugubris* [23], *Cladonia metacorallifera* [24], and *Usnea aurantiaco-atra* [25], which, along with phenolic compounds (depsides, depsidones, tridepsides, dibenzofurans, anthraquinones, chromones, and xanthones), contain various polyhydroxylated lipids (azelaic acid, hydroxy fatty acids) and wedelolactone in significant concentrations that increase the chemical fingerprint’s heterogeneity and enhance synergistic activity against therapeutic targets. Furthermore, metabolomics studies of Antarctic lichen species have enriched the literature data on secondary metabolites reported in Chilean species [26] and broadened the chemical spectrum, contributing to an increase in studies that elucidate their mechanisms of action at the cellular and molecular levels and their potential pharmacological effects.

### 2.2. Total Phenolic Content and Antioxidant Activity

Table 2 details the results of the assays for total phenolic content (TPC) and antioxidant activity for the hydroalcoholic extract of *L. puberulum*. Regarding TPC, the value represents a low-to-moderate concentration of phenolic compounds with 6.356 ± 0.15 mg GAE/g extract and considering the extraction yield of 5% (*w*/*w*), this corresponds to 0.318 mg GAE/g of dry lichen thallus; however, biological activity may be enhanced by the synergy of all detected compounds, such as aromatics, organic acids, and lipids. Regarding the reduction of Fe (III) metals by FRAP and free radical scavenging by ORAC, the hydroalcoholic extract reported values of moderate significance, with 21.925 ± 0.44 and 15.845 ± 1.633 µmol Trolox/g extract, respectively.

In the DPPH• assay, the extract exhibited an IC_50_ value of 1187.149 ± 12.5 µg/mL, and in the ABTS•^+^ assay, a value of 207.001 ± 0.006 µg/mL, compared to the gallic acid and quercetin standards, which indicates weak antioxidant potential and demonstrates the variability of this activity in Antarctic lichens [18,19,20,21,22,23,24,25]; furthermore, this limited radical scavenging activity is directly related to the low concentration of phenolic compounds (6.356 mg GAE/g) identified in the extract.

This activity is also comparable to that of other studied lichens with higher TPC values and similar radical activity, which have different geographic distributions and are of great biological and medical importance. This is the case for species such as *Ramalina farinacea*, *Pseudevernia furfuracea* [27], *Evernia prunastri* [27,28], *Pseudocyphellaria compar*, *Pseudocyphellaria nudata* [29], *Stereocaulon tomentosum*, *Lobaria pulmonaria*, *Cetraria islandica*, *Umbilicaria hirsuta*, *Xanthoria elegans* [30], and *Usnea lethariiformis* [31], among others, which exhibit variable but significant antioxidant potential and contribute considerably to the evaluation of lichens as sources of bioactive compounds with therapeutic effects. Furthermore, recent research in green chemistry [32,33] allows for the consideration of new sustainable extraction methods that enhance the antioxidant activity of lichen extracts and promote the chemical study and utilization of natural resources.

It is important to note that the analyses of chemical composition, phenolic compounds, and antioxidants were performed on a sample representing a single biological replicate (*n* = 1), in order to ensure that a sufficient and standardized extract was obtained for the experimental procedure. Although measurements were taken in triplicate, there is a limitation in not accounting for the chemical variability that the species *L. puberulum* may exhibit at the intra-thalline and inter-population levels. Therefore, these results reflect the specific profile of the sampled population, and it is justified, in future studies, to expand the geographic sampling to highlight the intrinsic chemical variations and similarities of the species.

### 2.3. In Silico Pharmacokinetic Profiling Based on Lipinski and Veber Rules

The evaluation of the pharmacokinetic properties of absorption, distribution, metabolism, and excretion (ADME) of secondary metabolites obtained from the lichen *L. puberulum* was based on the Lipinski and Veber rules, which allowed for distinguishing between metabolites with a favorable pharmacokinetic profile and those with some structural violations. The classic Lipinski (MW ≤ 500 Da, ≤5 H-bond donors, ≤10 acceptors, log *p* ≤ 5), and Veber (TPSA ≤ 140 Å^2^ and ≤10 rotatable bonds) rules are widely accepted for predicting the oral bioavailability of small organic molecules, although their application to highly structural natural products has known limitations [34].

The results of the in silico pharmacokinetic analysis showed that the low molecular weight aromatic compounds (benzoic acid, wedelolactone, and sinapoyltartronic acid) met all the parameters established in the Lipinski and Veber rules (Table 3). The polyhydroxylated fatty acids (PHTA, THDA, and THHA) showed a high number of hydrogen bond donors for the PHTA compound, as well as a high number of rotatable bonds for all three fatty acids (PHTA, THDA, and THHA). However, these compounds exhibited topological polar surface area (TPSA) values within a moderate range (118–138 Å^2^), resulting in an oral absorption rate between 61% and 68% (Table 3).

These results demonstrate that, although their passive permeability across biological membranes may be lower than that of conventional drugs, their physicochemical properties are within a threshold compatible with moderate absorption and bioavailability potential. This bioavailability could be improved in physiological environments involving active transport mechanisms or interactions with transport proteins [35].

It is important to recognize that Lipinski’s rule of five was originally derived from databases of orally administered synthetic drugs; therefore, it may not fully reflect the bioavailability behavior of highly oxygenated natural products. Polyhydroxylated fatty acids, such as PHTA, THDA, THHA, and THTA, deviate structurally from conventional drug-like molecules because they possess long aliphatic chains and a high degree of hydroxylation, which increase the number of rotating bonds and hydrogen bond donors beyond classical thresholds [36]. However, these structural features are characteristic of endogenous lipid mediators, such as eicosanoids and hydroxylated fatty acids, which are absorbed and distributed via active transport systems, including fatty acid transport proteins (FATPs) and albumin-mediated circulation, rather than relying solely on passive transcellular diffusion [37]. Consequently, strict application of Lipinski’s rule may underestimate the actual oral bioavailability of these compounds, and the calculated %ABS values (61–68%) should be interpreted as conservative estimates rather than definitive predictions for this type of natural product [35].

Therefore, based on these pharmacokinetic results for the secondary metabolites of *L. puberulum*, those organic molecules that did not exhibit more than one violation of the Lipinski and Veber rule were selected for further toxicological analysis. Consequently, the molecules GLHA, M14O, PHHA, and PHOA were excluded from the subsequent phases of the computational analysis (Table 3).

It is important to mention that, in the context of a lichen extract, oral administration may not be the primary route for these compounds. While the calculated %ABS values (61–68%) suggest moderate oral absorption potential, polyhydroxylated fatty acids of this structural class can also be administered via alternative routes, depending on the target pathology and formulation strategy. The physicochemical profile of PHTA, THDA, THHA, and THTA—moderate lipophilicity (cLogP 4.25–5.16), long aliphatic chains, and TPSA in the range of 118.22–138.45 Å^2^—is compatible with topical and transdermal administration, as these parameters favor partitioning in lipid formulations and permeation through the lipid layers of the skin [38]. Its structural similarity to endogenous lipids of the skin barrier further supports its potential use in topical anti-inflammatory preparations. Furthermore, for pathologies affecting mucosal surfaces, ophthalmic or intranasal routes could be considered, given that fatty-acid-derived compounds with similar TPSA ranges have demonstrated mucosal bioavailability [39]. Regarding PHTA specifically, its TPSA of 138.45 Å^2^, while slightly below Veber’s threshold of 140 Å^2^, does not preclude bioavailability via alternative routes, as topical and mucosal permeability is governed primarily by the partition coefficient and membrane affinity, rather than strict rules of similarity to oral drugs [40]. Therefore, the oral bioavailability estimates presented here should be interpreted as one pharmacokinetic dimension among several, and future formulation studies aimed at topical, transdermal, or mucosal administration would be pharmacologically aligned with both the biological origin of these metabolites and the anti-inflammatory applications of interest.

### 2.4. Computational Toxicity Assessment of Identified Metabolites

**Figure 4:**
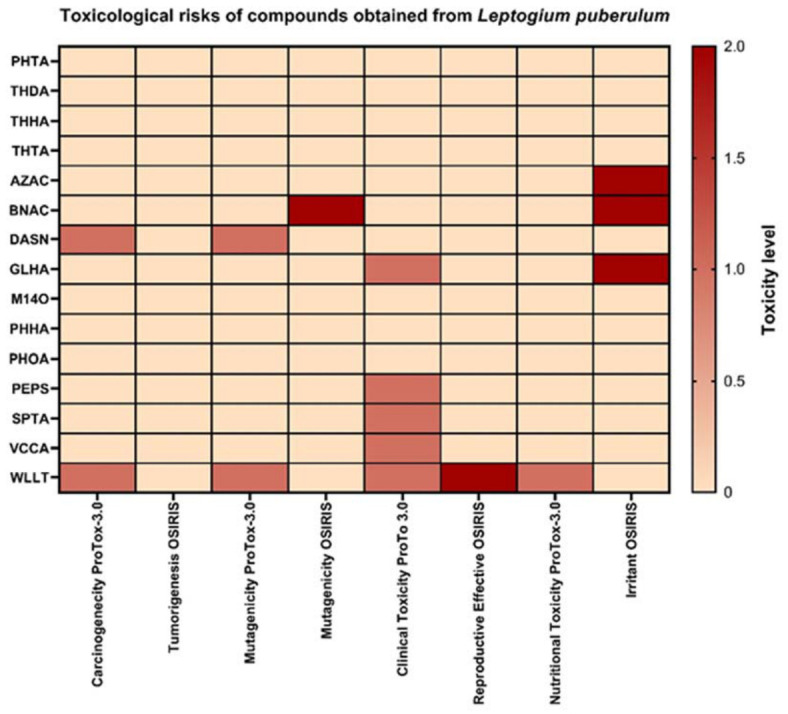
Evaluation of carcinogenic, mutagenic, reproductive system, and irritant effects of compounds obtained from *L. puberulum* using two different computer programs (ProTox 3.0 and OSIRIS). Note: PHTA: 9,10,12,13,14-pentahydroxytetracosanoic acid; THDA: 9,10,12,13-tetrahydroxydocosanoic acid; THHA: 9,10,12,13-tetrahydroxyheneicosanoic acid; THTA: 9,10,12,13-tetrahydroxytricosanoic acid; AZAC: azelaic acid; BNAC: benzoic acid; DASN: 1,3-diaceto-2-stearin; GLHA: gleditschiaside A; M14O: methyl 9,10,11,12,13-pentahydroxy-14-oxoheptacosanoate; PHHA: pentahydroxyhexacosanoic acid; PHOA: pentahydroxyoxohexacosanoic acid; PEPS: phenylethyl primeveroside; SPTA: Sinapoyltartronic acid; VCCA: vaccihein A y WLLT: wedelolactone.

Figure 4 presents a heat map summarizing the toxicological predictions obtained by the OSIRIS DataWarrior and Protox 3.0 software programs for all identified compounds. The color scale ranges from 0 to 2, where values of 0 (light colors) indicate no predicted toxicity for a given parameter, values of 1 (intermediate colors) indicate moderate predicted toxicity, and values of 2 (dark colors) indicate high predicted toxicity. This scoring system is derived from the structural alert-based toxicity prediction models in each software program, which evaluate each compound against a set of parameters including mutagenicity, tumorigenicity, irritant potential, and reproductive toxicity. Compounds exhibiting predominantly light coloration (score = 0 for most parameters) were considered toxicologically favorable and were prioritized for subsequent molecular docking analysis. Conversely, compounds that had scores of 1 or 2 on one or more parameters were excluded from further pharmacological evaluation, regardless of their pharmacokinetic profiles, since structural toxicity alerts represent a primary criterion for compound disqualification in the early stages of drug discovery [41].

The results of the toxicological evaluation of secondary metabolites of *L. puberulum* showed that polyhydroxylated fatty acids and other long-chain aliphatic compounds (PHTA, THDA, THHA, THTA, PHHA, and PHOA) did not present any carcinogenic or mutagenic risk on any of the in silico prediction platforms (Figure 4). This behavior suggests that their moderate lipophilicity, along with a low density of electrophilic functional groups, limits their ability to form covalent adducts with biological macromolecules, thus reducing their genotoxic potential. However, it is important to note that, according to the results obtained from the pharmacokinetic evaluation (Table 3), the compounds PHHA and PHOA exhibited low oral bioavailability, due to multiple violations of the Lipinski–Veber rules.

The compound BNAC showed a high mutagenicity signal according to the OSIRIS prediction, while AZAC showed a higher irritant potential. Furthermore, the compounds GLHA, PEPS, SPTA, and VCCA showed intermediate clinical toxicity values predicted by ProTox 3.0, suggesting potential adverse effects associated with high doses or prolonged exposures (Figure 4). The compound WLLT presented the greatest toxicity risks on both the OSIRIS and ProTox 3.0 platforms; moderate to high signals were observed in the carcinogenicity, mutagenicity, and reproductive toxicity categories (Figure 4). This behavior suggests that greater structural complexity, in combination with the presence of potentially reactive functional groups, could increase the likelihood of adverse interactions at the molecular level.

Integrating the toxicological profile results (Figure 4) with the pharmacokinetic evaluation (Table 3) allowed the identification of the polyhydroxylated fatty acids PHTA, THDA, THHA, and THTA as the main candidates for molecular docking evaluation, predictively assessing the potential therapeutic targets of these selected compounds.

### 2.5. Prediction of Molecular Targets Associated with Polyhydroxylated Fatty Acids

The prediction of potential molecular targets for the polyhydroxylated fatty acids identified in *L. puberulum* (PHTA, THDA, THHA, and THTA) was performed using the SwissTargetPrediction computational platform (Table 4). This tool bases its predictions on the principle of structural similarity and probabilistic models trained with a wide range of bioactive molecules [42,43]. The results obtained from the prediction of polyhydroxylated fatty acids showed that the prostaglandin E_2_ receptor, subtype EP_2_ (PTGER2), is one of the therapeutic targets with the highest probability of interaction for these compounds (Table 4). This association is mainly attributed to the lipophilic characteristics of these metabolites and the presence of oxygenated functional groups in their structure, elements that act as endogenous ligands of the prostaglandin pathway.

Predictive analysis using SwissTargetPrediction identified other potential targets; however, PTGER2 consistently ranked as the most likely target among the four polyhydroxylated fatty acids (Table 4). Furthermore, PTGER2 plays a significant role in COX-2-dependent inflammatory signaling as the primary receptor for PGE2, justifying the prioritization of this target [44]. This finding suggests that polyhydroxylated fatty acids (PHTA, THDA, THHA, and THTA) could modulate prostaglandin E_2_ (PGE_2_)-mediated signaling, a pathway of recognized importance in the regulation of inflammatory, proliferative, and neuroimmunological processes.

Since EP_2_ receptor activation depends directly on PGE_2_ availability, and since PGE_2_ biosynthesis is regulated by the cyclooxygenase-2 (COX-2) enzyme, it was decided to complement the target prediction with a molecular docking study specifically targeting COX-2. This approach allows us to assess whether polyhydroxylated fatty acids could interact directly with the enzyme responsible for producing the endogenous ligand PTGER2, thus exploring a possible regulatory mechanism step upstream of the pathway [45]. It is important to clarify that COX-2 inhibition, rather than direct coupling of PTGER2, is part of a deliberate upstream modulation strategy. Since PTGER2 is activated by PGE2, whose biosynthesis depends on the catalytic activity of COX-2, COX-2 inhibition reduces PGE2 production and, consequently, attenuates EP2 receptor activation, achieving indirect modulation of the PTGER2-mediated inflammatory pathway [32]. This mechanism is well supported by the pharmacological rationale for Non-Steroidal Anti-Inflammatory Drugs (NSAIDs). Direct docking of PTGER was not attempted because although a cryo-electron microscopy (cryo-EM) structure of the EP2–Gs complex (PDB: 7CX2) is available, this protein is transmembrane and its active conformation introduces significant complexity to standard molecular docking protocols [46].

The in silico evaluation of secondary metabolites identified in *L. puberulum* established a coherent framework linking their pharmacokinetic properties, toxicological profile, and potential molecular mechanism of action. This multidimensional approach allows for a preliminary visualization of the behavior of these organic molecules and facilitates their acceptance in the early stages of drug discovery, as it enables the prioritization of compounds with the highest probability of biological success and lowest risk of toxicity [47,48].

From a pharmacokinetic perspective, most of the analyzed secondary metabolites exhibited physicochemical properties consistent with good bioavailability, including moderate lipophilicity, molecular weights within acceptable ranges, and a polarity that favors passive permeability. The fact that these compounds exhibited no or at least one violation of the Lipinski and Veber rules suggests that they possess a suitable balance between solubility and permeability, a determining factor in oral absorption [40,49]. These characteristics are particularly relevant in oxygenated lipid metabolites, whose ADME behavior is often influenced by their structural similarity to endogenous mediators.

In accordance with these pharmacokinetic results, in silico toxicological analysis revealed a favorable safety profile for most of the metabolites, with a low or negligible probability of carcinogenicity, mutagenicity, and reproductive toxicity. However, only the polyhydroxylated fatty acids (PHTA, THDA, THHA, THTA) exhibited acceptable pharmacokinetic and toxicological parameters. This behavior can be attributed to the limited presence of highly reactive electrophilic groups and the aliphatic nature of many of the compounds, which reduces their potential for covalent interaction with critical biological macromolecules such as DNA or regulatory proteins [50].

The prediction made with SwissTargetPrediction indicated the prostanoid receptor EP_2_ (PTGER2) as one of the most likely therapeutic targets for polyhydroxylated fatty acids. This prediction is based on structural similarity to known bioactive ligands and has demonstrated high reliability for identifying relevant targets in natural product studies [42,43]. PTGER2 is a G-protein-coupled receptor activated by prostaglandin E_2_ (PGE_2_) and plays a fundamental role in inflammatory, neuroinflammatory, and proliferative processes [44].

The biological relevance of PTGER2 is reinforced by its close functional relationship with the cyclooxygenase-2 (COX-2) enzyme, responsible for the biosynthesis of PGE_2_ from arachidonic acid. In this context, molecular docking studies targeting COX-2 allow for the exploration of indirect modulation of PTGER2-mediated signaling. Inhibition or modulation of COX-2 leads to a decrease in PGE_2_ levels and, consequently, to a reduction in the activation of EP receptors, including EP_2_ [45,51].

### 2.6. Molecular Docking and Molecular Dynamics of Bioactive Compounds

The polyhydroxylated fatty acids (PHTA, THDA, THHA, THTA) together with celecoxib and ibuprofen were evaluated as ligands in molecular docking studies using the entire binding cavity of COX-2 (PDB ID: 5F1A). AutoDock Vina was employed to explore optimal binding conformations, generating multiple protein–ligand complexes. The theoretical affinities of protein–ligand complexes are reported as mean values ± standard deviation (kcal/mol) in Table 5. The redocking validation procedure employed is described in the Methods section, leading to a value of RMSD 1.387 ± 0.035 Å.

Overall, celecoxib exhibited the most favorable predicted binding affinity (−8.603 ± 0.814 kcal/mol) statistically, consistent with its role as a selective COX-2 inhibitor. Among the tested compounds, PHTA showed the strongest binding (−7.569 ± 0.124 kcal/mol), followed by THHA and THDA, which displayed comparable affinities. THTA presented the least favorable binding energy within the series.

Notably, ibuprofen showed an affinity (−7.188 ± 0.021 kcal/mol) similar to several of the evaluated ligands, serving as a useful reference for non-selective COX inhibition.

**Figure 5:**
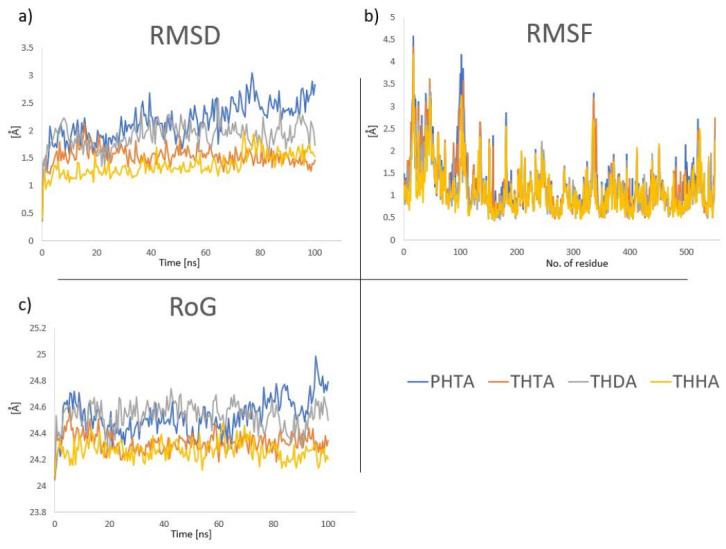
(**a**) Root Mean Square Deviation (RMSD); (**b**) Root Mean Square Fluctuation (RMSF); (**c**) radius of gyration (RoG) after 100 ns of MD. Note: PHTA: 9,10,12,13,14-pentahydroxytetracosanoic acid; THDA: 9,10,12,13-tetrahydroxydocosanoic acid; THHA: 9,10,12,13-tetrahydroxyheneicosanoic acid; THTA: 9,10,12,13-tetrahydroxytricosanoic acid.

These docking results provide an initial ranking of ligand affinity; however, the lower theoretical affinities protein–ligand complexes of each compound were selected to perform 100 ns MD studies. The structural stability of the complexes during MD simulations was assessed by backbone RMSD, residue-based RMSF, and radius of gyration (RoG) analyses (Figure 5a–c).

After 100 ns of molecular dynamics, the analyses of the trajectories reveal some key features. First, THHA and THTA complexes exhibited the highest structural rigidity. THHA showed the lowest mean RMSD (1.37 ± 0.20 Å), followed closely by THTA (1.54 ± 0.17 Å). Their RMSF values (1.11 Å and 1.08 Å, respectively) indicate that these ligands effectively restrict backbone fluctuations. THDA complex displayed moderate stability, with an RMSD of 1.91 ± 0.24 Å and a mean RMSF of 1.23 Å, suggesting a stable binding mode that allows limited induced-fit adaptations within the binding pocket. PHTA complex was the most dynamic, presenting the highest RMSD (2.17 ± 0.36 Å) and pronounced local flexibility (maximum RMSF of 4.57 Å), indicating substantial conformational sampling to maintain binding.

On the other hand, the compactness of complexes was evaluated through RoG analyses, finding that all complexes maintained a stable radius of gyration ranging from 24.27 Å to 24.53 Å, confirming that none of the ligands induced protein unfolding or significant loss of globular compactness. Interestingly, although AutoDock Vina (kcal/mol) and YASARA (kJ/mol) employ different scoring functions and energy units, their combined analysis revealed a notable re-ranking of ligand affinities following MD simulations. In YASARA protocols, the more positive the binding energy, the more favorable the interaction, as is explained and used in the literature (see Section 3.6.4).

PHTA initially appeared as the most favorable ligand based on docking scores (−7.569 kcal/mol). However, after 100 ns of MD simulation, THDA emerged as the most stable binder, exhibiting the highest YASARA binding energy (157.95 kJ/mol). In contrast, THTA displayed a negative binding energy in YASARA (−44.63 kJ/mol), indicating that its docking pose was energetically metastable and became unfavorable upon equilibration, in concordance with the observed value of −6.965 kcal/mol obtained in Viña. On the other hand, the well-known selective COX-2 inhibitor celecoxib affords an LBE value of 33.938 after 100 ns of MD simulation, evaluated in the same conditions as PHTA, THDA, THHA, and THTA. As can be noted, THDA and PHTA emerged as the more stable protein–ligand complexes, even better than celecoxib, emerging as candidates for further investigations.

The differences between docking ranking versus MD ranking could be explained by two approaches: in the first approach, while PHTA exhibited the most favorable AutoDock Vina score, docking calculations inherently treat the receptor as largely rigid and do not account for explicit solvent effects or long-timescale conformational flexibility. In contrast, molecular dynamics simulations revealed that PHTA does not maintain its initial docking pose over time. In this first explanation, trajectory analysis indicates that PHTA undergoes progressive displacement from the cyclooxygenase channel, accompanied by loss of key stabilizing interactions. In particular, hydrogen bonding and π-related contacts with residues such as Tyr385, His386, and His207 are not consistently preserved, leading to increased positional fluctuation and reduced binding stability.

By comparison, THDA maintains a more stable interaction network throughout the simulation, preserving persistent contacts with these residues and exhibiting lower structural drift within the binding channel. This results in a more favorable post-MD binding energy despite its slightly weaker docking score.

**Figure 6:**
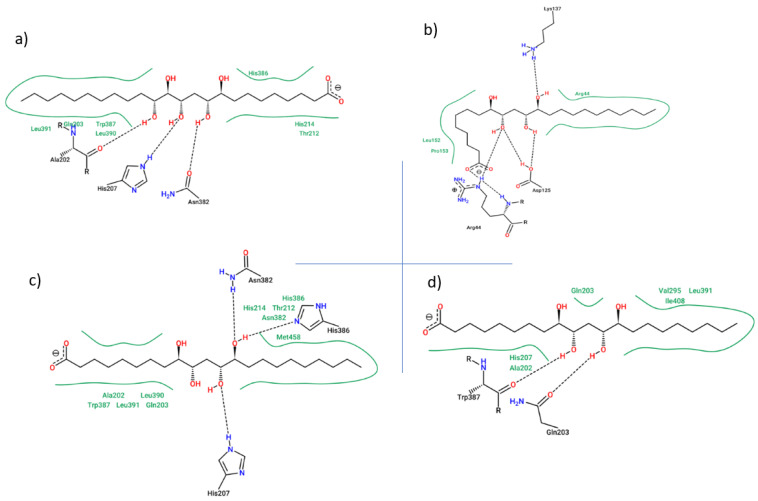
2D diagrams of (**a**) THDA; (**b**) PHTA; (**c**) THTA; (**d**) THHA. Note: PHTA: 9,10,12,13,14-pentahydroxytetracosanoic acid; THDA: 9,10,12,13-tetrahydroxydocosanoic acid; THHA: 9,10,12,13-tetrahydroxyheneicosanoic acid; THTA: 9,10,12,13-tetrahydroxytricosanoic acid.

The second approach to explaining energetic trends observed is through the analysis of the specific binding regions targeted by each ligand within the COX-2 enzyme. The binding mode THDA within the COX-2 active site reveals a functionally relevant interaction pattern that supports its inhibitory potential (Table 6 and Figure 6a). THDA establishes a stable hydrophobic interaction with Tyr385, the key catalytic residue responsible for hydrogen abstraction during the cyclooxygenase reaction. Occupation of this region is critical, as direct engagement with Tyr385 is a hallmark of effective COX-2 inhibition and is required for disruption of the catalytic radical mechanism.

In addition to hydrophobic packing against Tyr385, it forms hydrogen bond interactions with both His386 and His207, suggesting a dual anchoring mechanism along the catalytic axis and the access region of the enzyme. The hydrogen bond with His386, which lies immediately adjacent to Tyr385 within the catalytic core, contributes to precise ligand positioning and stabilization of the catalytic microenvironment. This interaction is particularly relevant, as perturbation of the His386–Tyr385 region is known to directly impair cyclooxygenase activity [52,53].

The interaction with His207, located in the lobby region of COX-2, appears to further enhance binding persistence by providing an additional polar anchor during ligand accommodation and entry into the catalytic channel. While His207 does not directly participate in catalysis, its involvement may facilitate proper ligand orientation and retention, thereby supporting sustained engagement with the catalytic site [54].

The binding mode of PHTA within the COX-2 active site reveals extensive ligand–protein interactions distributed along the catalytic channel and its entrance region. PHTA establishes multiple hydrophobic contacts with Leu390, Leu391, and Trp387, indicating that the ligand is able to penetrate the deep catalytic cavity. These residues are known to contribute to the hydrophobic environment required for substrate accommodation and inhibitor binding, suggesting that PHTA reaches a functionally relevant region of the enzyme.

However, several PHTA interactions involves residues with polar side chains, including Gln203, Thr212, Thr206, and His207, which are primarily located in the lobby and transitional regions of the COX-2 binding pocket. The ligand forms an extensive hydrogen-bonding network with Thr206, His207, Thr212, Thr383, and Tyr385, reflecting a binding mode that relies heavily on polar interactions rather than on deep hydrophobic packing resulting in reduced energetic stability. Although PHTA engages the catalytic residue Tyr385 through hydrogen bonding, this interaction differs mechanistically from the hydrophobic packing compared to THDA (Table 6 and Figure 6b).

Despite its high structural stability (lowest RMSD), THHA exhibited low binding affinity (+34.97 kJ/mol). The ligand binds predominantly to a distal aliphatic region (Val444, Val295, and Leu294), distant from the catalytic core, rendering it structurally stable but functionally non-inhibitory (Figure 6c). Finally, THTA failed to achieve favorable binding energy in YASARA due to its localization within the membrane binding domain (MBD) and lobby entrance. Interactions with residues such as Arg44, Arg61, and Asn43 kept the ligand solvent-exposed and prevented penetration into the hydrophobic catalytic channel (Figure 6d).

Collectively, the interaction profile of THDA indicates that the ligand successfully bridges the entrance and the deep catalytic region of COX-2, combining initial recognition with effective catalytic site blockade. The simultaneous engagement of Tyr385 through hydrophobic interactions and the formation of stabilizing hydrogen bonds with His386 and His207 suggests a binding mode consistent with a high-affinity, functionally competent COX-2 inhibitor. This cooperative interaction network likely contributes to the favorable energetic and structural stability observed during molecular dynamics simulations and supports the ligand’s potential as a lead compound for further optimization, compared with lipids and commercial selective and non-selective COX-2 inhibitors [55].

**Figure 7:**
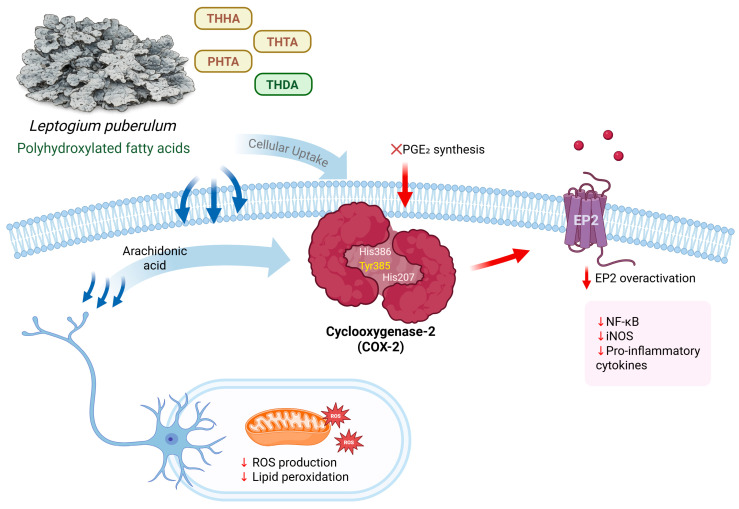
Proposed hypothetical mechanism of action of polyhydroxylated metabolites derived from *L. puberulum* (created in Biorender.com). This mechanism is based solely on computational predictions (molecular coupling and molecular dynamics simulations) and represents a working hypothesis pending experimental validation through in vitro and in vivo studies. Note: THDA = 9,10,12,13-tetrahydroxydocosanoic acid; PHTA = 9,10,12,13,14-pentahydroxytetracosanoic acid; THHA = 9,10,12,13-tetrahydroxyheneicosanoic acid; THTA = 9,10,12,13-tetrahydroxytricosanoic acid; COX-2 = cyclooxygenase-2; PGE_2_ = prostaglandin E_2_; EP2 = prostaglandin E receptor subtype 2; ROS = reactive oxygen species.

Based on the results of the antioxidant potential of the extract and the in silico analysis of the molecular interactions of compounds, we propose a hypothetical mechanism of action involving cell signaling, antioxidant activity, and neuroprotection for the polyhydroxy metabolites derived from *L. puberulum* (Figure 7). The evaluated compounds that can enter the cell via fatty acid transport proteins (FATPs) or facilitated diffusion, particularly THDA, interact with the catalytic site of cyclooxygenase-2 (COX-2), establishing stable interactions with key residues such as Tyr385, His386, and His207, resulting in a modulation of prostaglandin E_2_ (PGE_2_) synthesis. The reduction of PGE_2_ levels leads to decreased activation of the prostanoid EP2 receptor (PTGER2), regulating inflammatory and neuroimmune signaling pathways associated with reactive oxygen species (ROS) production. This modulation contributes to the attenuation of neuroinflammation, reduction of oxidative stress, and promotion of an antioxidant and neuroprotective cellular state.

Finally, based on the work conducted and the results presented and discussed, it is essential to acknowledge a methodological limitation regarding the anti-inflammatory activity of the *L. puberulum* extract, given that the findings are based exclusively on computational predictions using docking and molecular dynamics analyses. While the chemical characterization and antioxidant capacity were validated in vitro, the interaction of the four polyhydroxylated fatty acids with the COX-2 enzyme and the subsequent modulation of PGE2 synthesis were explored via in silico analysis as a strategy for prioritization and preliminary visualization in preclinical trials to elucidate the biological potential of the compounds present in lichens.

These tools allowed us to propose a hypothetical mechanism in which the catalytic site of COX-2 can be inhibited through stable interactions with critical residues such as Tyr385. However, this working hypothesis will be addressed in future studies through enzyme inhibition assays and cell line tests that will allow us to evaluate the effect on inflammatory pathways and validate the efficacy suggested by theoretical models.

## 3. Materials and Methods

### 3.1. Lichen Material

A total of 100 g of *L. puberulum* lichen thallus growing on small rocks on the ground was collected by A.T.-B. on Livingston Island, located in the South Shetland Islands archipelago of Maritime Antarctica, in February 2025. The species were identified using specialized identification keys based on macroscopic and microscopic morphological characteristics, and the updated nomenclature was verified using online databases and scientific literature. Specimens of each species were deposited at the Natural Products Laboratory of the Institute of Pharmacy at the Universidad Austral de Chile (Valdivia, Chile) under identification number HL-01042025.

### 3.2. Preparation of Hydroalcoholic Extract

Using 10 g of *L. puberulum*, maceration was performed with a 70:30 (*v*/*v*) methanol–water mixture in an ultrasonic bath (80 kHz) at 35 °C, with three extractions using 50 mL of solution every 30 min. Subsequently, the total hydroalcoholic extract was filtered and concentrated under reduced pressure in a rotary evaporator at 38 °C until a gummy consistency was obtained. The yield of the extract was 5.0%.

### 3.3. LC Parameters and MS Parameters

Chemical profiling was conducted using an ultra-high-resolution liquid chromatography system coupled with time-of-flight mass spectrometry using electrospray ionization and a quadrupole (UHPLC-ESI-QToF-MS). The instrumentation comprised an Ultimate 3000 RS UHPLC (Dionex GmbH, Idstein, Germany) controlled by Chromeleon 6.8 software, coupled with a Bruker DataAnalysis 4.0 maXis ESI-QToF-MS (Bruker Daltonik GmbH, Bremen, Germany). For the analysis, 5 mg of each extract was dissolved in 2 mL of analytical-grade methanol, followed by filtration through polytetrafluoroethylene (PTFE) membranes. A 10 μL volume was injected into the chromatographic system, which was equipped with a quaternary pump, an automatic injector, a thermostated column compartment, and a diode array detector (DAD). Separation of the analytes was performed on a Thermo C18 column (150 mm × 4.6 mm, 5 µm, 80 Å) at a constant flow rate of 1.0 mL/min. Elution was performed using a binary gradient consisting of 0.1% formic acid in water (phase A) and 0.1% formic acid in acetonitrile (phase B): isocratic at 1% B (0–2 min); gradient from 1 to 5% B (2–3 min); isocratic at 5% B (3–5 min); 5–10% B (5–8 min); 10–30% B (8–30 min); 30–95% B (30–38 min); and finally, an isocratic phase at 1% B (39–50 min). ESI-QToF-MS measurements were performed in negative ionization mode with a scan range of 100 to 1200 *m*/*z*. The ESI source conditions were set to: 200 °C capillary temperature, 2.0 kV capillary voltage, 8 L/min drying gas flow, and 2 bar nebulizer pressure. The structural elucidation of the compounds was based on exact mass (HR-MS), the fragmentation patterns obtained, and thorough comparison with data reported in the literature. Furthermore, since absolute stereochemistry cannot be determined solely by MS/MS, no isolations were performed, nor were chiral standards used; the compounds identified were described using the most common configurations reported in Antarctic lichen species.

### 3.4. Total Phenolic Content

The total phenolic content (TPC) was determined using the Folin–Ciocalteau (FC) colorimetric method [56]. Gallic acid was used as a standard, and a calibration curve was prepared. Ten milligrams of extract were weighed and dissolved in 1 mL of buffer to prepare four dilutions. Each sample and standard concentration was mixed with 12.5 µL of FC and 150 µL of distilled water, incubated at 40 °C for 5 min, 37.5 µL of Na_2_CO_3_ was added, incubated for 30 min at 40 °C, and finally read at 765 nm. The results were expressed in mg of gallic acid equivalents per g of extract.

### 3.5. Determination of Antioxidant Properties

Free radical scavenging capacity was determined using the 2,2-diphenyl-1-picrylhydrazyl (DPPH•) assay [57]. A 200 µM DPPH• solution was prepared by dissolving the compound in absolute ethanol. Quantification was performed using a calibration curve of gallic acid as the standard compound. A total of 2.5 mg of extract was weighed, and 1 mL of methanol was added. The mixture was then sonicated for 1 min at 37 °C to ensure complete dissolution. Serial dilutions were prepared using this stock solution. Each standard and sample concentration was placed in triplicate in a 96-well microplate along with 150 µL of DPPH•. The reaction was incubated in the dark for 30 min, and the absorbance was measured at 517 nm. The final results were expressed in µg/mL, reporting the half-maximal inhibitory concentration (IC_50_).

The ABTS•^+^ radical cation assay was performed using 2,2′-azino-bis(3-ethylbenzothiazoline-6-sulfonic acid) (ABTS•^+^) [58]. A stock solution of the compound was prepared using Na_2_S_2_O_8_ and distilled water; the mixture was incubated in the dark at room temperature for 24 h, and the ABTS•^+^ stock solution was subsequently diluted with ethanol. Trolox was used as the standard, and a calibration curve was prepared. A stock solution was prepared from 10 mg of extract, and serial dilutions were made. In a 96-well microplate, 50 µL of sample and standard were added in triplicate, along with 150 µL of ABTS•^+^ solution. The reaction was then incubated for 30 min in the dark, and the absorbance was measured at 732 nm. The final results were expressed in µg/mL, reporting the half-maximal inhibitory concentration (IC_50_).

The ferric reduction antioxidant power (FRAP) assay involved the reduction of the ferric complex of 2,4,6-tripyridyl-s-triazine (from Fe^3+^-TPTZ to Fe^2+^-TPTZ) [59]. A calibration curve was prepared using the Trolox standard, and dilutions were prepared. A total of 10 mg of extract was weighed and dissolved in 1 mL of acetate buffer, and serial dilutions were prepared. In a 96-well microplate, 10 µL of the standard and sample concentrations were added in triplicate; 290 µL of FRAP reagent was added; the plate was incubated at room temperature for 60 min in the dark; and the absorbance was measured at a wavelength of 593 nm. The results are expressed in µmol Trolox/g of extract.

The oxygen radical absorbance capacity (ORAC) assay was based on the generation of free radicals through the oxidation of 2,2′-azobis(2-methylpropionamidine) dihydrochloride (AAPH) using a fluorescent probe such as fluorescein [60]. An 18 mM AAPH solution was prepared with 25 mg in 5 mL of PBS, and a 108 nM fluorescein solution was prepared. Trolox was used as the standard for the calibration curve. A total of 5 mg of extract was weighed and dissolved in 1 mL of PBS buffer. In a 96-well microplate, 50 µL of each standard and sample concentration was added in triplicate; then 195 µL of fluorescein was added, the mixture was incubated for 30 min at 37 °C, and 50 µL of AAPH was added; subsequently, readings were taken every 2 min for 2 h at an excitation wavelength of 480 nm and an emission wavelength of 520 nm. The results are expressed in µmol Trolox/g of extract.

### 3.6. In Silico Analysis

#### 3.6.1. Calculation of Pharmacokinetic Parameters (ADME)

To evaluate the pharmacokinetic properties of secondary metabolites obtained from the lichen *L. puberulum*, the OSIRIS Data Warrior (v 5.5.0) computational platform was used. The calculated molecular descriptors are based on the Lipinski–Veber rules and include the logarithm of the partition coefficient (cLogP), the number of hydrogen bond donors, the number of hydrogen bond acceptors, the molecular mass of the compounds, the topological polar surface area (TPSA), and the number of rotatable bonds. Based on the results obtained, compounds that did not exhibit more than one violation of the Lipinski–Veber rule were evaluated. The results obtained for the topological polar surface area allowed for the calculation of the percentage absorption (%ABS), which was calculated using the following Equation (1) [18,40,49]:

%ABS = 109 − (0.345 × TPSA)(1)

#### 3.6.2. Toxicity Risk Assessment and the Prediction of Bioactivity

To calculate the possible toxicological risks of the secondary metabolites obtained from *L. puberulum*, the OSIRIS Data Warrior and ProTox 3.0 computational platforms were used. The toxicity risks that were evaluated were mutagenicity, tumorigenicity, carcinogenicity, irritation, clinical toxicity, nutritional toxicity, and reproductive effects [18]. For the evaluation of the bioactivity of compounds that exhibited good pharmacokinetic properties and no or low toxicological risk, the prediction of potential therapeutic targets for viable compounds from *L. puberulum* was performed using the online platform SwissTargetPrediction.

#### 3.6.3. Molecular Docking

An in silico molecular docking study was performed to evaluate the theoretical binding capacity of a set of compounds against the cyclooxygenase-2 (COX-2) enzyme (PDB ID: 5F1A, resolution 2.38 Å) [61]. Ligands 9-10-12-13-14-pentahydroxytetracosanoic acid (PHTA), 9-10-12-13-tetrahydroxytricosanoic acid (THTA), 9-10-12-13-tetrahydroxyheneicosanoic acid (THHA), and 9-10-12-13-tetrahydroxydocosanoic acid (THDA), celecoxib and ibuprofen were constructed, polar hydrogens were added, and their three-dimensional structures were energy-minimized using the MMFF94 force field with MolConvert (ChemAxon, 2024; Marvin/MolConvert, version 24.1.0) and saved in *.pdbqt format.

The protein structure was obtained from the RCSB Protein Data Bank (RCSB.org) [62]. Protein preparation for docking was performed using AutoDockTools (ADT) version 1.5.7 [63]. Crystallographic water molecules and artifacts were removed, Gasteiger charges were assigned, and non-polar hydrogens were merged. Protonation states of basic and acidic amino acid residues were adjusted to physiological pH (7.4).

The docking grid box was defined as a cube with side lengths of 30 Å, centered at X = 41.62, Y = 24.07, and Z = 240.26, which corresponds to a cube centered on resolved co-crystallized salicylic acid retained around the active site of the enzyme after acetylation of Ser530 by aspirin. Molecular docking simulations were carried out using AutoDock Vina with an exhaustiveness value of 64 in triplicate [64]. Docking protocol validation was conducted by redocking salicylic acid into its corresponding active site using the same docking parameters in triplicate. The resulting poses of salicylic acid conformers were compared with the crystallographic conformation by calculating the Root Mean Square Deviation (RMSD) using PyMOL v2.5.0, yielding an RMSD value of 1.387 ± 0.035 Å. The four protein–ligand complexes with the lowest predicted binding affinities were selected for subsequent molecular dynamics (MD) simulations using YASARA.

#### 3.6.4. Molecular Dynamics Simulations

Molecular dynamics (MD) simulations of 100 ns were performed for each selected protein–ligand complex using YASARA software, following a previously described protocol [65]. Briefly, each complex was positioned in a water box with a size of 100 Å × 100 Å × 100 Å, with periodic boundary conditions (PBC). The temperature was set at 298 K, and the water density was set at 0.997 g/cm^3^. The sodium (Na^+^) and chlorine (Cl^−^) ions were included for neutralization and to provide conditions that simulate a physiological solution (pH 7,4, NaCl 0.9%). The particle–mesh Ewald (PME) algorithm with a cut-off radius of 8 Å was applied. The simulation snapshots were recorded at intervals of 250 ps with a time step of 2.5 fs until a total simulation time of 100 ns was reached. After completion of the simulations, the resulting trajectories were analyzed using YASARA’s built-in tools, including RMSD, Root Mean Square Fluctuation (RMSF), and radius of gyration (RoG) variations over time as well as ligand binding energy (LBE) calculations using the MM-PBSA method. LBE calculations were performed on snapshots from the last 75 ns of the simulations.

In YASARA protocols, the more positive the binding energy, the more favorable the interaction [66]. Finally, the average structures of each complex were extracted and analyzed using PLIP (Protein–Ligand Interaction Profiler) [67] and the ProteinsPlus PoseView server [68] to obtain two-dimensional interaction diagrams and interaction profiles.

### 3.7. Statistical Analysis

In the molecular docking experiments, each measurement was performed in triplicate and expressed as mean + standard deviation (SD). To evaluate the statistical significance of the binding affinities obtained from the molecular docking simulations, a one-way ANOVA was performed, followed by Tukey’s post hoc multiple comparison tests (*p* < 0.05). Starting with a single standardized extract, the in vitro assays, each measurement was performed in three independent experiments, each carried out in triplicate, and the data were organized in Microsoft Excel 2019 and expressed as mean + standard deviation (SD). For the comparison of means, a one-way ANOVA analysis of variance was performed, and considering a *p*-value < 0.05, significant differences were established with the Tukey test, using GraphPad Prism 8 software.

## 4. Conclusions

This study represents the first report on the chemical composition and biological potential of the Antarctic lichen species *L. puberulum*. Through UHPLC/ESI/QToF/MS analysis of the hydroalcoholic extract, 18 secondary metabolites were tentatively identified, including long-chain polyhydroxy fatty acids (PHTA, THDA, THHA, and THTA). Furthermore, in vitro assays of phenolic content and antioxidant activity revealed results of low-to-moderate significance compared to other reported Antarctic lichen species; however, this highlights the wide variability that can be observed in the study of lichen extracts and suggests that their biological relevance does not strictly depend on nonspecific antioxidant potential. Regarding the in silico analysis, the pharmacokinetic variables suggest a favorable profile for the oral bioavailability of the evaluated compounds present in the *L. puberulum* extract. Likewise, the docking and molecular dynamics models indicate a theoretical affinity of the fatty acids for the catalytic site of the COX-2 enzyme.

These results allow us to propose a hypothesis regarding the possible modulation of prostaglandin E2 (PGE2) synthesis; however, this interaction should be considered a preliminary prediction due to the absence of specific enzymatic and/or cellular assays to evaluate this therapeutic target. In general, *L. puberulum* represents a potential source of bioactive compounds for the study of inflammatory diseases; however, biological validation through cellular and animal models is necessary to confirm the proposed mechanism of action, determine efficacy and safety parameters, and strengthen the future health applications that lichen-derived metabolites may have.

## Figures and Tables

**Table 1:** Identification of the compounds present in the hydroalcoholic extract of *L. puberulum* through UHPLC/ESI/QToF/MS.

Peak	Retention Time (min)	Tentative Identification	[M-H]^−^	Theoretical Mass (*m*/*z*)	Measured Mass (*m*/*z*)	Accuracy (ppm)	Metabolite Type	MS Ions (ppm)
1	0.37	Na formiate (internal standard)	C_4_H_2_O_4_	112.9829	112.9856	3.1	-	---
2	1.34	Mannitol	C_6_H_13_O_6_	181.0712	181.0705	3.9	C	151.0598
3	1.78	Citric acid	C_6_H_7_O_7_	191.0192	191.0184	4.2	OA	111.0074
4	1.89	Vaccihein A	C_18_H_17_O_9_	377.0819	377.0814	−1.35	A	---
5	12.92	Benzoic acid	C_7_H_5_O_2_	121.0295	121.0271	5.3	A	111.0796
6	14.76	Azelaic acid	C_9_H_15_O_4_	187.0975	187.0968	−0.9	L	179.0330, 125.0976
7	15.23	Sinapoyltartronic acid	C_14_H_13_O_9_	323.04623	323.04637	−1.11	A	187.09352, 204.02216, 160.03604
8	16.45	Wedelolactone	C_16_H_9_O_7_	313.0295	313.0308	4.37	A	269.03821
9	20.23	1,3-Diaceto-2-stearin	C_23_H_45_O_6_	433.3117	433.3144	−6.0	L	277.2144
10	20.87	9,10,12,13-tetrahydroxyheneicosanoic acid	C_21_H_41_O_6_	389.2903	389.2892	2.8	L	371.2784
11	21.12	9,10,12,13,14-pentahydroxytetracosanoic acid	C_24_H_47_O_7_	447.3322	447.3306	3.6	L	389.2891, 429.3199, 361.2581
12	21.45	9,10,12,13-tetrahydroxydocosanoic acid	C_22_H_43_O_6_	403.3060	403.3047	3.2	L	385.2939, 215.1273
13	21.89	Pentahydroxyoxohexacosanoic acid	C_26_H_49_O_8_	489.3432	489.3403	−5.9	L	403.3001, 979.6848 (2M-H)
14	23.04	9,10,12,13-tetrahydroxytricosanoic acid	C_23_H_45_O_6_	417.3236	417.3204	5.7	L	297.23287
15	23.56	Phenylethyl primeveroside	C_19_H_27_O_10_	415.1609	415.1654	5.6	A	297.2365, 163.0348
16	24.54	Methyl 9,10,11,12,13-pentahydroxy-14-oxoheptacosanoate	C_28_H_53_O_8_	517.3740	517.3719	4.1	L	457.3510, 439.3404
17	25.12	Pentahydroxyhexacosanoic acid	C_26_H_51_O_7_	475.3635	475.3618	3.6	L	---
18	26.08	Gleditschiaside A	C_21_H_27_O_11_	455.15591	455.1558	5.12	A	409.0848, 327.24549
19	28.94	Unknown	C_7_H_15_O_13_	307.05181	307.05494	−1.26	-	263.06370

Note: A = aromatic; C = carbohydrate; OA = organic acid; L = lipid.

**Table 2:** Total phenolic content (TPC) and antioxidant activity of the hydroalcoholic extract of lichen *L. puberulum*.

Assay	TPC mg GAE/g	FRAP µmol Trolox/g	ORAC µmol Trolox/g	DPPH IC_50_—µg/mL	ABTS IC_50_—µg/mL
*L. puberulum*	6.356 ± 0.15 *	21.925 ± 0.44 *	15.845 ± 1.633 *	1187.149 ± 12.5 *	207.001 ± 2.1 *
Gallic acid ^#^	-	-	-	2.24 ± 0.04 *	16.5 ± 0.04 *
Quercetin ^#^	-	-	-	12.25 ± 0.6 *	15.65 ± 0.05 *

Values marked with * are statistically different (*p* < 0.05). ^#^ Positive controls.

**Table 3:** Evaluation of the pharmacokinetic properties of the secondary metabolites isolated from the lichen *L. puberulum* according to the rules of Lipinski and Veber.

ID Code	Compound	Structural Formula	Violation of Lipinski’s Rule					Violation of Veber’s Rule			
			MW ^a^ <500	cLogP ^b^ ≤5	HBD ^c^ ≤5	HBA ^d^ ≤10	Violations ≤1	*n*-ROTB ^e^ ≤10	TPSA (Å^2^) ^f^ ≤140	%ABS ^g^	Violations ≤1
PHTA	9,10,12,13,14-pentahydroxy tetracosanoic acid	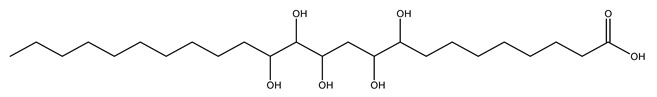	448.64	4.59	6	7	1	22	138.45	61.23	1
THDA	9,10,12,13-tetrahydroxy docosanoic acid	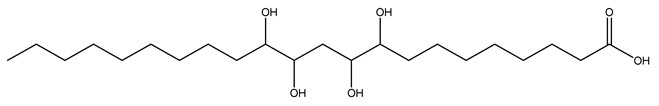	404.59	4.70	5	6	0	20	118.22	68.21	1
THHA	9,10,12,13-tetrahydroxy heneicosanoic acid	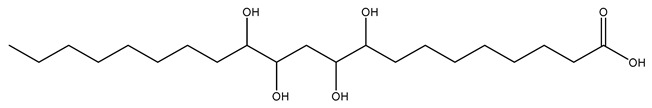	390.56	4.25	5	6	0	19	118.22	68.21	1
THTA	9,10,12,13-tetrahydroxy tricosanoic acid	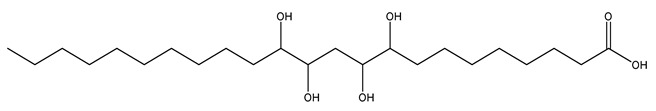	418.61	5.16	5	6	1	21	118.22	68.21	1
AZAC	Azelaic acid	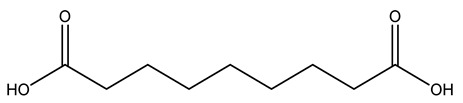	188.22	1.61	2	4	0	8	74.60	83.26	0
BNAC	Benzoic acid	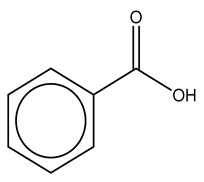	122.12	1.44	1	2	0	1	37.30	96.13	0
DASN	1,3-Diaceto-2-stearin	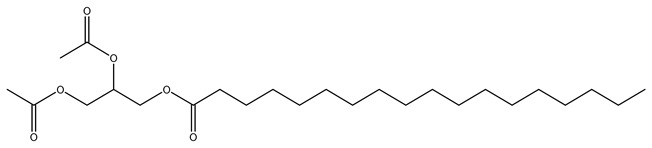	442.63	7.28	0	6	1	24	78.90	81.78	1
GLHA	Gleditschiaside A	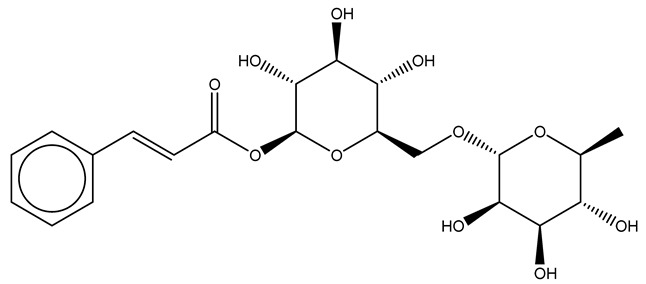	456.44	−1.27	6	11	2	7	175.37	48.50	1
M14O	Methyl 9,10,11,12,13-pentahydroxy-14-oxoheptacosanoate	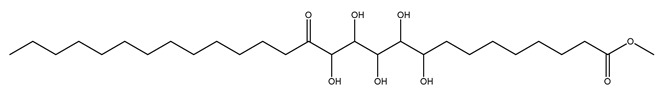	518.73	5.33	5	8	2	26	144.52	59.14	2
PHHA	Pentahydroxy hexacosanoic acid	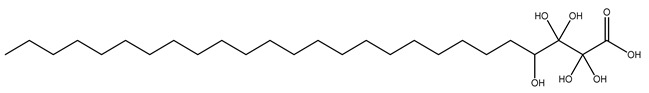	476.69	7.29	6	7	2	24	138.45	61.23	1
PHOA	Pentahydroxy oxohexacosanoic acid	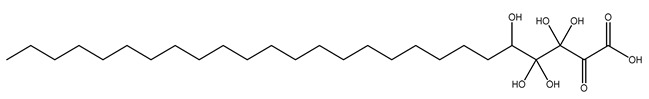	490.68	6.24	6	8	2	24	155.52	55.35	2
PEPS	Phenylethyl primeveroside	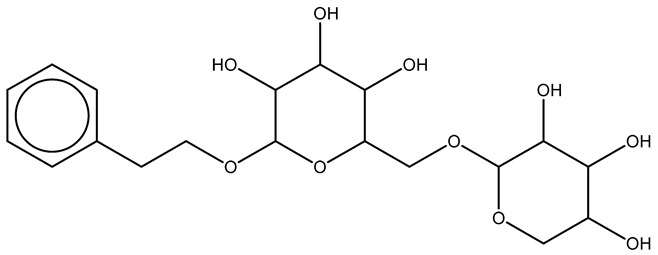	416.42	−1.58	6	10	1	7	158.30	54.39	1
SPTA	Sinapoyltartronic acid	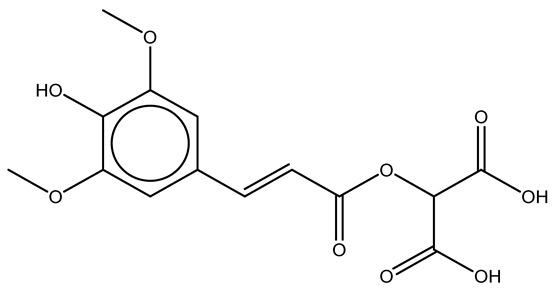	326.26	−0.37	3	9	0	8	139.59	60.84	0
VCCA	Vaccihein A	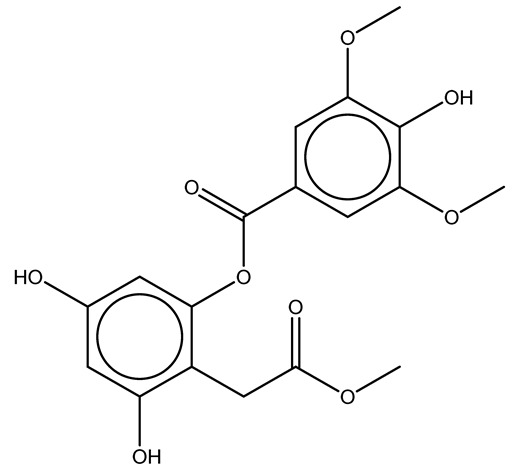	378.33	1.82	3	9	0	8	131.75	63.55	0
WLLT	Wedelolactone	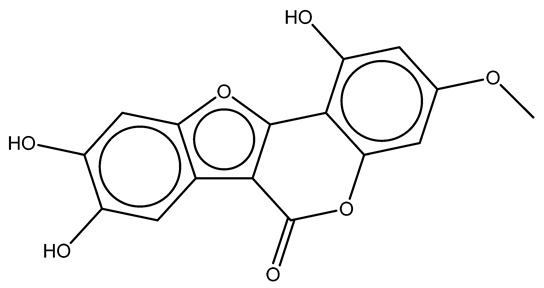	314.25	2.43	3	7	0	1	109.36	71.27	0

Note: PHTA: 9,10,12,13,14-pentahydroxytetracosanoic acid; THDA: 9,10,12,13-tetrahydroxydocosanoic acid; THHA: 9,10,12,13-tetrahydroxyheneicosanoic acid, THTA: 9,10,12,13-tetrahydroxytricosanoic acid; AZAC: azelaic acid; BNAC: benzoic acid; DASN: 1,3-diaceto-2-stearin; GLHA: gleditschiaside A; M14O: methyl 9,10,11,12,13-pentahydroxy-14-oxoheptacosanoate; PHHA: Pentahydroxyhexacosanoic acid; PHOA: Pentahydroxyoxohexacosanoic acid; PEPS: Phenylethyl primeveroside; SPTA: sinapoyltartronic acid; VCCA: vaccihein A y WLLT: wedelolactone. ^a^ molecular weight (MW); ^b^ logarithm of partition coefficient between n-octanol and water (cLogP); ^c^ number of hydrogen bond donors (HBD); ^d^ number of hydrogen bond acceptors (HBA); ^e^ number of rotable bonds (n-ROTB); ^f^ topological polar surface area (TPSA); ^g^ percentage of absorption (%ABS).

**Table 4:** Predicted molecular targets of *L. puberulum* metabolites using SwissTargetPrediction.

ID Code	Target	Common Name	Uniprot ID	Target Class	Probability
PHTA	Prostanoid EP2 receptor	PTGER2	P43116	Family A G protein coupled receptor	0.199
THDA	Prostanoid EP2 receptor	PTGER2	P43116	Family A G protein coupled receptor	0.304
THHA	Prostanoid EP2 receptor	PTGER2	P43116	Family A G protein coupled receptor	0.257
THTA	Prostanoid EP2 receptor	PTGER2	P43116	Family A G protein coupled receptor	0.174

Note: PHTA: 9,10,12,13,14-pentahydroxytetracosanoic acid; THDA: 9,10,12,13-tetrahydroxydocosanoic acid; THHA: 9,10,12,13-tetrahydroxyheneicosanoic acid; THTA: 9,10,12,13-tetrahydroxytricosanoic acid.

**Table 5:** Values of binding affinities calculated in AutoDock Vina and YASARA software after 100 ns of simulation.

Ligand	Vina Docking Theoretical Affinities (kcal/mol)	YASARA Post-MD LBE (kJ/mol)
THDA	−7.149 ± 0.27 ^bc^	+157.951
PHTA	−7.569 ± 0.124 ^bc^	+135.171
THHA	−7.241 ± 0.351 ^c^	+34.969
THTA	−6.965 ± 0.168 ^b^	−44.632
Celocoxib	−8.603 ± 0.814 ^a^	+33.938
Ibuprofen	−7.188 ± 0.021 ^bc^	ND

Note: PHTA: 9,10,12,13,14-pentahydroxytetracosanoic acid; THDA: 9,10,12,13-tetrahydroxydocosanoic acid; THHA: 9,10,12,13-tetrahydroxyheneicosanoic acid, THTA: 9,10,12,13-tetrahydroxytricosanoic acid. ND: not determined. Note: means within the same column sharing at least one letter in the “Tukey Significance Group” do not differ significantly from each other according to Tukey’s HSD test (*p* > 0.05).

**Table 6:** Key interactions between ligands and COX-2 enzyme.

Ligands		Residues with Key Interactions
THDA	HI	Tyr148, Ala202, Thr212, Ala379, Asn382, Tyr385, Trp387, Leu390, Leu391
	HB	Thr206, His207, Asn382, His386
PHTA	HI	Gln203, Thr212, Trp387, Leu390, Leu391
	HB	Thr206, His207, Thr212, Thr383, Tyr385
THTA	HI	His39, Arg44, Arg61, Tyr130, Pro153
	HB	Cys41, Asn43, Arg44, Thr129, Lys137
THHA	HI	Ala202, Gln203, Phe210, Leu294, Val295, Tyr404, Ile408, Val444
	HB	Trp387

Note: PHTA: 9,10,12,13,14-pentahydroxytetracosanoic acid; THDA: 9,10,12,13-tetrahydroxydocosanoic acid; THHA: 9,10,12,13-tetrahydroxyheneicosanoic acid; THTA: 9,10,12,13-tetrahydroxytricosanoic acid. HI: hydrophobic interactions at max. of 4.0 Å of distance of carbon atoms for a hydrophobic interaction; HB: hydrogen bond at max. of 4.1 Å of distance between acceptor and donor.

## Data Availability

The datasets presented in this study can be consulted with the authors by correspondence.
